# Supplementary material for: Intelligence against complexity: Machine learning for nonuniform temperature-field measurements through laser absorption
Source: PLoS One. 2022 Dec 12;17(12):e0278885. doi: 10.1371/journal.pone.0278885 (PMC9744279; doi:10.1371/journal.pone.0278885)
Supplement: Attachment — Submitted filename: Response to Reviewers-PONE-D-22-24085.pdf [file pone.0278885.s001.pdf]

Response to the Editor and Reviewers

First of all, we would like to express our sincere thanks to the editor and reviewer, who helped us in improving both the quality and presentation of this manuscript, by providing valuable suggestions to improve our work. In the following sections, we will first reply to the requirements/comments from the editor and proceed with the reply to the comments from the reviewer.

Response to Journal Requirements

J1. Please ensure that your manuscript meets PLOS ONE's style requirements, including those for file naming. The PLOS ONE style templates can be found at https://journals.plos.org/plosone/s/file?id=wjVg/PLOSOne_formatting_sample_main_body.pdf and https://journals.plos.org/plosone/s/file?id=ba62/PLOSOne_formatting_sample_title_authors_affiliations.pdf

Response: We would like to confirm that the PLOS ONE's official LaTex format template was used in the preparation of this manuscript.

J2. Please note that PLOS ONE has specific guidelines on code sharing for submissions in which author-generated code underpins the findings in the manuscript. In these cases, all author-generated code must be made available without restrictions upon publication of the work. Please review our guidelines at https://journals.plos.org/plosone/s/materials-and-software-sharing#loc-sharing-code and ensure that your code is shared in a way that follows best practice and facilitates reproducibility and reuse.

Response: We would like to thank PLOS ONE for this reminder. As originally stated, at the point of submission, we made all related ***code and data*** available on Github at ***https://github.com/RalphKang/nonuniformity-effect-on-LAS--temperature-measurement***. Alternatively, one can check the release of the repository through doi: 10.5281/zenodo.7303621. Moreover, as a complement to the code, we uploaded the dataset and pretrained models on Kaggle, available through ***https://www.kaggle.com/datasets/ralphkang/laser-absorption-spectra-of-co2***. In both the readme.md of the repository and description of the dataset, all necessary information and descriptions were provided to allow replication of results/reuse of models.

J3. Thank you for stating the following financial disclosure: "DCK would like to acknowledge partial support by Khalifa University through grant RC2-2019-009". Please state what role the funders took in the study. If the funders had no role, please state: "The funders had no role in study design, data collection and analysis, decision to publish, or preparation of the manuscript." If this statement is not correct you must amend it as needed. Please include this amended Role of Funder statement in your cover letter; we will change the online submission form on your behalf.

Response: We would like to thank you for this helpful comment. The appropriate statement is "The

funders had no role in study design, data collection and analysis, decision to publish, or preparation of the manuscript".

J4. Thank you for stating the following in the Acknowledgments Section of your manuscript: "DCK would like to acknowledge partial support by Khalifa University through grant RC2-2019-009". We note that you have provided funding information that is not currently declared in your Funding Statement. However, funding information should not appear in the Acknowledgments section or other areas of your manuscript. We will only publish funding information present in the Funding Statement section of the online submission form. Please remove any funding-related text from the manuscript and let us know how you would like to update your Funding Statement. Currently, your Funding Statement reads as follows: "DCK would like to acknowledge partial support by Khalifa University through grant RC2-2019-009". Please include your amended statements within your cover letter; we will change the online submission form on your behalf.

Response: Thank you for your clarification. The acknowledgment of partial financial support for this research was removed, and instead, the first author provides an academic acknowledgement. The removed funding statement was has been included in the cover letter for this revision.

J5. Thank you for stating the following in your Competing Interests section: "NO authors have competing interests". Please complete your Competing Interests on the online submission form to state any Competing Interests. If you have no competing interests, please state "The authors have declared that no competing interests exist", as detailed online in our guide for authors at http://journals.plos.org/plosone/s/submit-now This information should be included in your cover letter; we will change the online submission form on your behalf.

Response: Thank you for this clarification. We included the statement that "No authors have competing interests" in the cover letter, and would be grateful if could update the online submission form on our behalf.

J6. We note that you have stated that you will provide repository information for your data at acceptance. Should your manuscript be accepted for publication, we will hold it until you provide the relevant accession numbers or DOIs necessary to access your data. If you wish to make changes to your Data Availability statement, please describe these changes in your cover letter and we will update your Data Availability statement to reflect the information you provide.

Response: Please see our response under point **J2** as follows: As originally stated, at the point of submission, we made all related ***code and data*** available on Github at ***https://github.com/RalphKang/nonuniformity-effect-on-LAS--temperature-measurement***.

Alternatively, one can check the release of the repository through doi: 10.5281/zenodo.7303621. Moreover, as a complement to the code, we uploaded the dataset and pretrained models on Kaggle, available through ***https://www.kaggle.com/datasets/ralphkang/laser-absorption-spectra-of-co2***. In both the readme.md of the repository and description of the dataset, all necessary information and descriptions

were provided to allow replication of results/reuse of models.

J7. Please review your reference list to ensure that it is complete and correct. If you have cited papers that have been retracted, please include the rationale for doing so in the manuscript text, or remove these references and replace them with relevant current references. Any changes to the reference list should be mentioned in the rebuttal letter that accompanies your revised manuscript. If you need to cite a retracted article, indicate the article's retracted status in the References list and also include a citation and full reference for the retraction notice.

Response: We carefully reviewed the list of references and can confirm that there are no instances of references being retracted. Moreover, the details of all cited works are complete.

Response to the comments of the reviewer

R1.C1: This an outstanding paper, written with high quality style and good English language. The authors have compared between sixteen models for uniform-profile spectra and the best three models, i.e., GPR, VGG and BRF were further compared for non-uniform-profile data. The paper is well-documented and well strutured and presented in a clear manner. The results are deeply analyzed. I have no comment about the paper, except one minor 1. I wonder if the authors can provide a shoot discussion, more precisely, a comparison of the present paper with other reported studies in the literature before the conclusion.

Response: We are sincerely grateful to the reviewer for his encouraging comments and for recognizing the contributions of our work. We fully agree with the reviewer's suggestion and in this respect, we incorporated a new section entitled "Discussion" as follows:

"In this work, we provided quantitative results and associated discussions in terms of the effect of spatial non-uniformity with respect to the parameters of temperature and concentration distribution on the accuracy of the average temperature measurement estimate using laser absorption spectroscopy. The focus of the research was to explore the feasibility whether machine learning methods could provide reliable parameter estimates by overcoming this kind of effect. Indeed, some earlier studies [11], [13], [15] provided the means to measure average temperature from absorption spectra subject to the presence of non-uniformity. However, they neither provided a systematic study of the effects of the non-uniformity, nor attempted to define its impact on the accuracy of the mean temperature measurements. For instance, in [12], there was an attempt to formally analyze and explain the mechanism of the effect of non-uniformities in temperature and distribution concentration. The outcomes of this work have merit, however, due to the complexities of the associated theoretical analysis in the simultaneous presence of non-uniformities in both temperature and concentration, the associated conclusions were not clear. In this research, we pursue a fundamentally different approach. The essence of the underlying concept is to use multiple machine-learning-based surrogate models, where each model was trained on uniform-profile data, to estimate the average temperature from nonuniform-profile data, and next, apply data analysis tools to quantify the effect of non-uniformity. Based on quantitative statistics, we confirmed that using temperature measurement tools for uniform profiles directly on spectra generated from non-uniform profiles introduces significant errors.

Lastly, to the best of our knowledge, this is the first work, which attempts to overcome non-uniformity effects in laser absorption spectra by machine-learning-based solutions. The results demonstrate that some machine-learning-based solutions, based on global information, demonstrate robustness to changes in the magnitude and style of non-uniformity in temperature and concentration distributions, which could potentially solve the problem of measuring average temperature from laser absorption spectra, in the case of nonuniform profiles."

[11] Tudor Palaghita and Jerry Seitzman. “Control of Temperature Nonuniformity Based on Line-of-Sight Absorption”. In: 40th AIAA/ASME/SAE/ASEE Joint Propulsion Conference and Exhibit. 2004, p. 4163. doi: 10.2514/6.2004-4163.

[12] Christopher S. Goldenstein et al. “Two-Color Absorption Spectroscopy Strategy for Measuring the Column Density and Path Average Temperature of the Absorbing Species in Nonuniform Gases”. In: Applied Optics 52.33 (2013), pp. 7950–7962. doi: 10.1364/AO.52.007950.

[13] Xiang Liu, Jay B. Jeffries, and Ronald K. Hanson. “Measurement of Nonuniform Temperature Distributions Using Line-of-Sight Absorption Spectroscopy”. In: AIAA Journal 45.2 (2007), pp. 411–419. doi: 10.2514/1.26708.

[15] Scott T. Sanders et al. “Diode-Laser Absorption Sensor for Line-of-Sight Gas Temperature Distributions”. In: Applied Optics 40.24 (2001), pp. 4404–4404. doi: 10.1364/ao.40.004404.
